# Exercise in Obesity—the Role of Technology in Health Services: Can This Approach Work?

**DOI:** 10.1007/s13679-021-00461-x

**Published:** 2021-11-17

**Authors:** Laurence J. Dobbie, Abd Tahrani, Uazman Alam, Jennifer James, John Wilding, Daniel J. Cuthbertson

**Affiliations:** 1 Department of Cardiovascular and Metabolic Medicine, Institute of Life Course and Medical Sciences, University of Liverpool, Liverpool, UK; 2 University Hospital Aintree, Liverpool University Hospitals NHS Foundation Trust, Liverpool, UK; 3 Institute of Metabolism and Systems, School of Clinical and Experimental Medicine, University of Birmingham, Birmingham, UK; 4 Department of Diabetes and Endocrinology, Birmingham Heartlands Hospital, Birmingham, UK

**Keywords:** Obesity, eHealth, Physical activity, Wearables, Exergaming

## Abstract

***Purpose of Review*:**

Physical activity (PA) is an important strategy to prevent and treat obesity. Electronic health (eHealth) interventions, such as wearable activity monitors and smartphone apps, may promote adherence to regular PA and successful weight loss. This review highlights the evidence for eHealth interventions in promoting PA and reducing weight.

***Recent Findings*:**

Wearables can increase PA and are associated with moderate weight loss in middle/older-aged individuals, with less convincing effects long-term (> 1 year) and in younger people. Data for interventions such as mobile phone applications, SMS, and exergaming are less robust. Investigations of all eHealth interventions are often limited by complex, multi-modality study designs, involving concomitant dietary modification, making the independent contribution of each eHealth intervention on body weight challenging to assess.

***Summary*:**

eHealth interventions may promote PA, thereby contributing to weight loss/weight maintenance; however, further evaluation is required for this approach to be adopted into routine clinical practice.

## Introduction

The past five decades has witnessed profound and continued evolution of the characteristics and quantities of food that individuals consume (particularly processed and ultra-processed food) and in the amount of physical activity (PA) which individuals undertake. A chronic mismatch between energy intake, with consumption of caloric excess/energy-dense foods, and energy expenditure, with increasing rates of physical inactivity, underlies the current obesity epidemic. Insufficient physical activity, also known as physical inactivity (defined as not undertaking at least 150 min of moderate-intensity, or 75 min of vigorous-intensity PA/week, or any equivalent combination of the two, including PA at work, at home, for transport, and during leisure time) has a global age-standardised prevalence of 27.5% (95% uncertainty interval 25.0–32.2) [1]. The prevalence of insufficient PA is highest and continually increasing in high-income countries, with women less active than men worldwide. Implementation of targeted evidence-based interventions, presented in the Global Physical Activity Action Plan 2018–2030, and publication of new Guidelines on Physical Activity and Sedentary Behaviour aim to improve population health by reversing the current trends and reducing disparities in physical activity [2, 3].

Current intervention strategies for prevention and treatment of obesity have significant limitations. The most effective strategy currently available for treating severe obesity is bariatric/metabolic surgery, but this may be associated with medical complications and weight regain and availability is limited [4]. Moderate-intensity regular PA alone is generally associated with moderate weight loss only, but still encouraging increased PA is an important strategy for optimising the effects of dietary modification and for weight loss maintenance [5–7]. Technology (computers, smartphones, internet usage, video gaming) has become embedded in our society with complex consequences for PA and obesity levels. Yet, ironically some of the same technological advances that have driven reductions in PA may also be adopted to facilitate increased levels of PA (and dietary compliance) for the prevention of obesity, as an effective adjunct to its treatment and in addressing obesity-related morbidities and for successful weight-loss maintenance [8].

### Benefits of Physical Activity

 The benefits of physical activity includes preventing or improving many chronic diseases and impacting on both all-cause mortality and life expectancy [9–11]. Wen et al*.* performed a prospective cohort study on 416,175 individuals in Taiwan from 1996 to 2008 to evaluate the impact of different levels of physical activity on all-cause mortality and life expectancy [12]. Individuals were placed into 5 categories: inactive, low, medium, high, and very high activity, according to their weekly exercise volume assessed using a self-administered questionnaire. The data highlighted that those in the low PA group, who exercised for an average of 15 min/day, had a 14% reduced risk of all-cause mortality and had a 3-year longer life expectancy, compared with those in the inactive group. Furthermore, each additional 15 min of exercise/day (beyond the minimum amount of 15 min/day) reduced all-cause mortality and all-cancer mortality by 4% and  1% respectively. These benefits were noted in both sexes, all ages and those with high cardiovascular disease (CVD) risk.

### Traditional and Novel Methods of Implementing Exercise Interventions

 Traditionally, exercise interventions have been supervised ‘in person’ on an individual or group basis; this pattern of intervention delivery is not feasible for large-scale public health interventions. The current coronavirus pandemic has impacted the way in which individuals engage in PA and has seen adoption of novel innovations to facilitate remote supervision and monitoring of PA. Strategies to enhance PA adherence and sustainability are associated with greater benefit. Increasingly, there has been interest in technology-based interventions to promote physical activity and health more remotely which have been shown to be effective in increasing PA levels [13]. These electronic Health (eHealth) interventions represent novel approaches in the promotion of PA, and reduction of body weight, and are vital given that ~ 50% of individuals attempting weight loss do not engage in any PA [14]. eHealth technologies include wearable devices, social networking sites (SNS), smartphone applications, short messaging service (SMS) and exergaming [13, 15–17]. For eHealth interventions to be implemented within obesity healthcare services, the technologies must be based on clinically proven behaviour change techniques (BCT), which are considered to be the ‘active ingredients’ in behaviour change interventions, with an increasing evidence base to support their use in the promotion of PA in clinical care [18].

Advances in digital technology and digital platforms can facilitate adherence to behavioural regimes and increase PA levels, optimising the process of self-monitoring (with automation of monitoring and feedback), enhancing self-efficacy and improving the motivation for behaviour change and, therefore, leading to greater success in long-term weight loss and maintenance [19]. Simultaneously, these technologies can be adapted to implement educational interventions and other changes in health behaviour such as dietary modification. Evidence to date suggests that electronic monitoring methods promote higher rates of adherence to self-monitoring than traditional paper-based methods [20–22]. This narrative review shall discuss the various modalities in which technology has been applied to enhance PA and exercise in individuals living with overweight and obesity.

## Wearables (Table 1)

Wearable technologies include smartwatches (e.g. Fitbit, Apple Watch) and provide continuous feedback on PA-relevant indices including step count, energy expenditure and heart rate [23]. The data generated facilitates goal setting, objective PA self-monitoring and social support via SMS which are evidence-based BCTs [20, 24, 25]. Automated self-monitoring aids may prompt identification of detrimental behaviour changes and overcomes significant limitations of paper monitoring such as imprecise recall and social desirability bias [15, 19, 21, 22, 26, 27]. Wearables have considerable potential for PA promotion when used alongside standard behavioural weight loss (SBWL) programmes [27, 28]. They also have potential to improve quality of life [29].

**Table 1:** Impact of wearable activity monitors on physical activity level and body weight

Paper	Details	Intervention	Study groups	Findings	Conclusion
*IDEA RCT* Jakicic et al*. *[42]	*N* = 471, 71.1%, female, BMI: 31.2 Age ~ 30.9 y	24 months Diet modification + PA increase ± wearable device	**2 groups** Standard: website monitoring diet/PA Enhanced: wearable + website monitoring	Standard: −5.9 kg* Enhanced: −3.5 kg (*p < 0.05 vs enhanced)	In young adults with overweight/obesity, wearable technology resulted in less weight reduction than SBWL intervention
*TRIPPA* Finkelstein et al*. *[34••]	*N* = 800, 53.8% Female, weight: ~ 66.1 kg, Age: ~ 35.5 y	12 months Wearable ± cash/charity incentive	**4 groups** Control Wearable: Fitbit activity monitor Cash + wearable Charity + wearable	Control: −22 MVPA, − 1.3 kg Wearable: +16 MVPA*, −0.4 kg Cash: +10 MVPA, − 0.8 kg Charity: −7 MVPA, −0.6 kg (MVPA = MVPA bout min per week, * = p < 0.05 vs baseline)	1 year of wearable brought significant increased PA, but no change in body weight
Cadmus-Bertram et al*. *[109]	*N* = 51, female, BMI: ~ 29.2 Age: ~60 y	16 weeks PA self-monitoring targeting 150 min/week MVPA	**2 groups** Web-based: Fitbit activity monitor Comparison: pedometer	Web-based: + 62 min/week MVPA*, −0.3 kg Comparison: + 13 min/week MVPA, + 0.01 kg (*p < 0.05 vs baseline)	Wearable associated with greater PA at 16 weeks. No change in weight but was not powered to detect this
Hartman et al*. *[25]	*N* = 54, female, elevated risk breast cancer, BMI: ~31.9 Age: ~ 59.5 y	6 months MyFitnessPal website/app to diet monitor + Fitbit activity monitor	**2 groups** Control: US dietary guidelines + 2 brief calls Intervention: MyFitnessPal + Fitbit activity monitor	Control: −0.5 kg, + 11 min/day MVPA Intervention: − 4.4 kg*, + 15 min/day MVPA (*p < 0.05 vs control)	Technology intervention promoting PA + DM reduced weight and brought non-significant increase in MVPA
Nicklas et al*. *[110]	*N* = 48, 75.5%, female BMI: 33.1 Age: ~ 70.1 y	5 months weight loss + 5 months follow-up Diet modification + PA ± activity monitor to prevent weight regain	**2 groups** Control: diet + PA Intervention: diet + PA + activity monitor	Control: − 5.0 kg, + 5.4 min/day LPA, + 5.7 min/day MVPA Intervention: − 8.6 kg*, + 5.7 min/day LPA, + 4.3 min/day MVPA (*p < 0.05 vs control)	Over 10 months wearable brought significantly greater WL; this was not driven by increased LPA/MVPA
Chen et al. [45]	*N* = 40, 42.5%, female BMI: 28.3 Age: ~ 14.9 y	6 months Wearable activity monitor + educational progression + SMS messages	**2 Groups** Control: pedometer + paper food/activity diary + online education modules Intervention: Fitbit activity monitor + online education modules + biweekly SMS + smartphone app tracking PA/diet	Control: BMI + 0.83, − 0.04 active days/week Intervention: BMI − 0.44*, + 0.73 active days/week (active day = > 60 min PA) (*p < 0.05 vs control)	Multi-modal intervention including wearable reduced BMI in adolescents. Did not demonstrate significant increase in PA days, however no objective measurement of PA
Thomas et al*. *[111]	*N* = 279, 77.5%, female BMI: ~ 33.9 Age: ~ 55.0 y	12 months Online programme with dietary/PA monitoring ± activity tracker	**3 groups** Control: newsletter Online: online program Monitor: online program + activity monitor	Control: + 5.3 min/day MVPA, − 601.9 kcal/day*, − 1.2 kg Online: − 2.4 min/day MVPA, − 472.9 kcal/day, − 2.1 kg Monitor: − 1.3 min/day MVPA, − 479.8 kcal/day, − 1.6 kg (*p < 0.05 vs monitor)	Following a 12-month intervention weight loss was not greater in an online program or activity monitor group than control

Original table created by authors

*BMI* body mass index, *y* years, *N* number, *PA* physical activity, *MVPA* moderately vigorous physical activity, *p* p-value, *SBWL* standard behavioural weight loss

### Influence of Wearables on PA

Wearable devices are an acceptable technology to a diverse range of populations [30–33]. The Singaporean TRIPPA study randomised 800 participants, aged 21–65 years, to four groups: a Fitbit activity tracker, tracker plus cash incentives, tracker plus charity incentive or control. Assessment of moderately vigorous physical activity (MVPA), the primary outcome measure, was made at intervention end, at 6 months and at 12 months (6 months post-intervention). At 6 months, the cash incentive was most effective, increasing MVPA by 29 bout min per week (95% CI 10–47; *p* = 0·0024). At 12 months, the activity tracker, with or without charity incentives, was effective at stemming the reduction in MVPA bout min/week observed within the control group, but there was no improvements in any health outcomes (weight or blood pressure) [34••].

Similarly, 51 inactive, overweight, post-menopausal women were randomised to activity monitor (Fitbit tracker) or standard pedometer (control). They demonstrated that activity monitoring significantly increased MVPA (62 ± 108 min/week (*p* < 0.01), and steps (by 789 ± 1979 (*p* = 0.01), over 16 weeks compared to non-significant increases in the control group. This pilot, however, was underpowered to detect between group differences [35].

In contrast, in Thompson et al.’s study, 49 older people (65–95 years) took part in a randomised controlled crossover study giving an accelerometer providing activity feedback with exercise counselling for 48 weeks versus no initial intervention and 24 weeks accelerometry/counselling. The eHealth intervention did not lead to significant changes in PA, body weight, percentage body fat or blood parameters (*p* > 0.05)[36]. Similarly, three further RCTs ((1) 104 medical doctors, (2) 50 middle aged men, (3) 227 Americans) showed that wearables do not significantly increase the PA level [37–39]. A systematic review of studies assessing wearable activity trackers suggested that they have the potential to increase PA participation as a primary component or as part of a broader intervention, but it acknowledged often only as short-term effects. However, to be implemented within health services, they must be shown to reduce weight [15].

### Influence of Wearables on Body Weight

Wearable activity monitors may enhance the effects of a SBWL intervention for weight reduction or even provide an equivalent substitute. Pellegrini et al. conducted a 3-arm intervention study in 51 people comparing an ‘in-person’ SBWL programme alone (SBWL), with a technology-based system (TECH) incorporating digital dietary and physical activity tracking capability (with weekly feedback based on these behaviours) with a combination of the two (SBWL + TECH). Body weight and physical activity were compared at baseline and after 6 months. The greatest weight loss at 6 months was seen with a combination of SBWL and TECH (− 8.8 ± 5.0 kg, − 8.7 ± 4.7%), lesser amounts with TECH alone (− 5.8 ± 6.6 kg, − 6.3 ± 7.1%) and the least with SBWL (− 3.7 ± 5.7 kg, − 4.1 ± 6.3%)(p <0.001). Self-reported PA increased significantly in all groups: SBWL (473.9 ± 800.7 kcal/week), SBWL + TECH (713.9 ± 1,278.8 kcal/week) and TECH (1,066.2 ± 1,371 kcal/week)(p<0.001). No between group differences were noted (*p* = 0.25) [40].

A larger RCT of 197 sedentary adults with overweight or obesity were randomised into 1 of 4 groups over 9 months: standard care (using a weight-loss manual), a group-based behavioural weight loss program (GWL), a SenseWear armband that tracks daily energy expenditure and energy intake (Armband) or a combination of the two (GWL + Armband). After 9 months, there was significant weight loss in all 3 intervention groups (GWL, 1.86 kg, *p* = 0.05; Armband alone, 3.55 kg, *p* = 0.0002; GWL + Armband, 6.59 kg, *p* < 0.0001) but not in the control group (0.89 kg, *p* = 0.39). Significant weight loss was only achieved at month 9 when comparing  the GWL + Armband group to  control  (*p* = 0.04) [41]. These investigations highlight the supplementary effect of PA monitoring when coupled with SBWL interventions on weight.

Wearable activity monitors may have greater efficacy in middle- to older-aged populations with some study results suggesting that activity monitoring is less effective in younger populations. In the IDEA RCT study of 471 young adults, aged 18–35 years, BMI 25–40 kg/m^2^, a technology-enhanced weight loss intervention (including a wearable activity monitor and web interface) resulted in less weight loss than a SBWL programme (3.5 vs 5.9 kg). However, significant improvements in body composition, fitness, PA, and diet were detected in both groups, with no significant difference between them [42]. The negative findings of the study may be confounded by wearable devices not being available from trial onset.

Similarly, the results of a smaller RCT of children with obesity, aged 10–17 years, comparing a 3-month standard weight loss intervention versus a personalised technology-based approach using a wristband to measure energy expenditure, a smartphone application to measure energy intake and weekly feedback, demonstrated equivalence [43]. However, in 48 older adults with obesity, age 65–79 years, randomised to a 5-month weight loss intervention of a hypocaloric diet, aerobic exercise with/without an accelerometer to provide real-time feedback and increase in PA/reduce sedentary time, the addition of the feedback resulted in greater weight loss and less weight regain than that seen in the control group [44].

The use of technology to implement multi-modal interventions promoting PA and dietary modification simultaneously make it difficult to independently assess their relative contributions to the weight loss. This is exemplified in an RCT of 54 women (at increased risk of breast cancer) comparing a 6-month SBWL program with a multi-modal technology-based intervention including activity monitoring (Fitbit), a mobile app (MyFitnessPal) and phone counselling. The multi-modal intervention group lost significantly more weight, despite similar PA levels, pointing to dietary modification rather than PA as the mediating factor [25]. Similar findings were seen in Chinese American adolescents [45].

A large network meta-analysis of 31 studies conducted in individuals with overweight/obesity reported that wearable-based interventions (using accelerometer, pedometer, or commercial devices) are effective interventions for reducing body weight and body mass index [46•]. Another meta-analysis also concluded similarly, suggesting that 12 weeks/more duration of intervention is more effective and that every week of wearable use reduces weight by 0.37% [47]. Regular activity monitoring is superior to intermittent use [48]. Evidence is strongest over the short and medium term; limited long-term efficacy data is available. Furthermore, data supports their efficacy in middle- to older-aged adults; younger people have a suboptimal response, and this requires further evaluation [15]. Complex study designs, involving multiple BCTs, make it challenging to determine whether wearables can independently influence weight [27].

### Reproducibility of Wearables Measurements

Multiple studies have determined the reproducibility and accuracy of wearable technologies for measurement of PA and associated energy expenditure (EE), an important consideration if these technologies are to be implemented in healthcare [13]. Accepting variable performances of different devices, step count measurement appears to be reproducible, but EE appears to be overestimated [49, 50]. Two systematic reviews also conclude that wearables provide reproducible measures of PA, but sub-optimal EE measurement [15, 51]. Thus, it appears that PA level or step count, rather than EE, can help participants to accurately monitor both progress and achievement of PA goals.

Overall, whilst wearable technologies can promote PA and weight loss in a healthcare setting, (Table 1); their independent effect on weight loss through increased PA is difficult to dissect from that of dietary modification. The devices show most promise in middle- to older-aged populations; younger populations may require alternative approaches, i.e. BCT. PA feedback data must be tailored to the patient group, ensuring it provides health behaviour insights without being overwhelmingly complex [15, 22, 23, 27, 52, 53]. Duration of use is also a consideration—most effective when used for at least 12 weeks—but longer-term efficacy is less clear [15, 47, 54]. Significant attrition does appear to be an issue. Services must ensure that the devices utilise evidence-based BCT and implement mechanisms ensuring long-term adherence given the documented > 30% attrition over 6 months and the need for sustained lifestyle changes [23].

## Mobile Phone Interventions (Table 2)

**Table 2:** Impact of smartphone applications on physical activity level and body weight

Paper	Details	Intervention	Study groups	Findings	Conclusion
*CITY Trial* Svetkey et al*. *[68]	*N* = 365, 69.6%, female BMI: ~ 35.2 Age: ~ 29.4 y	24 months Smartphone app ± personal coaching based on BCT	**3 groups** Control: weight loss hand-outs Smartphone: smartphone app BCT + self-monitoring Personal coaching: group BCT sessions + smartphone self-monitoring	Control: 12 m − 2.25 kg, 24 m − 1.44 kg Smartphone: 12 m − 1.48 kg, 24 m − 0.99 kg Personal coaching: 12 m − 3.58 kg* 24 m − 2.45 kg (*p < 0.05 vs smartphone)	Smartphone app with BCT and self-monitoring of diet and PA did not significantly reduce weight. Note does not report PA level
ENGAGED, Spring et al*. *[65]	*N* = 96, 84.4% Female BMI: ~ 34.6 Age: ~ 39.3 y	6 months with a 12-month follow-up Smartphone app with SNS features + accelerometry	**3 groups** Self-guided: paper monitoring diet/PA/weight Standard: paper monitoring + group BCT sessions Technology: smartphone app + group BCT sessions	Self-guided: 6 m − 2.7 kg, 12 m − 2.7 kg Standard: 6 m − 6.6 kg, 12 m − 5.6 kg Technology: 6 m − 4.7 kg, 12 m − 3.1 kg Standard + technology: 6 m − 5.7 kg*, 12 m − 4.4 kg (*p < 0.05 vs self-guided)	A technology intervention did not lead to significantly greater weight loss than self-guided intervention. Note that PA level was not reported on and that both PA + DM were used
Goldstein et al*. *[112]	*N* = 276, 83%, female BMI: ~ 35.2 Age: ~ 55.1 y	Post-hoc analysis of RCT Self-monitor diet, weight, PA via paper/smart-phone app DM + PA	**3 groups** Control = self-monitoring/feedback via paper diary Group = paper diary self-monitoring + group BWL Smart = smartphone self-monitoring and feedback	Group: *β* = 0.03* for PA predicting % weight loss, *β* = 0.21* for diet monitoring predicting % weight loss Smart: *β* = 0.02* for PA predicting % weight loss, *β* = 0.07 for diet monitoring predicting % weight loss (*p < 0.05)	Smartphone PA monitoring predicted weight loss to similar degrees as paper diary monitoring
Ryu et al*. *[107•]	*N* = 80, 23.5%, female BMI: ~ 27.5 Age: ~ 38.7 y	4 weeks Electronic health record tethered smartphone app monitoring diet + PA	**2 groups** Control Smartphone: tethered to electronic health record	Control: − 0.1 BMI Smartphone: − 0.5* BMI (*p < 0.05 vs baseline)	4 weeks of smartphone app linked to personal health record correlated with higher body weight loss. Note PA level not reported

Original table created by authors

*BMI* body mass index, *y* years, *N* number, *PA* physical activity, *MVPA* moderately vigorous physical activity, *p* p-value, *SBWL* standard behavioural weight loss

Mobile phone applications (apps) have been investigated for their role in monitoring PA and influencing PA interventions. Their varied functionalities include providing PA self-management information, facilitating self-monitoring via inbuilt accelerometers and linking with external devices. They provide reproducible PA measurements but require a strong evidence base before being implemented within a healthcare setting [55, 56].

### Effect of Mobile Apps on Daily Step Count

A variety of studies have suggested that mobile app interventions integrated with PA monitoring can increase step count [57–62]. In one large RCT of 200 people (50 intervention, 150 controls) in which a smartphone app was implemented within an existing eHealth intervention (10,000 steps Australia), researchers observed a greater odds of achieving > 10,000 steps/day [63]. Similarly in short-term (2 weeks) studies of sedentary females (*n* = 42) and longer-term studies (6 months) of patients with T2D (*n* = 12) smartphone apps increased step count by 800 steps and 1100 steps per day respectively [57, 64]. Overall, smartphone PA monitoring shows promise in promoting PA engagement and behaviour change when coupled with other PA interventions.

### Effects of Mobile Apps on Weight Loss

Smartphone apps integrated with pedometers are linked with weight loss [62, 65, 66]. In 61 patients with obesity at high risk of T2D, a standard intervention, based on the Diabetes Prevention Program, was compared with an intervention comprising reduced in person contact substituted with a combined mobile app and pedometer intervention to facilitate self-monitoring. Smartphone self-monitoring (*n* = 30) was associated with greater weight loss than the control intervention (weight change: − 6.2 vs + 0.3 kg) and higher PA levels (+ 2551 vs − 734 steps/day) [58].

Similar to the phenomenon observed with wearables, smartphone app/mobile interventions have a less pronounced effect in younger populations. In a 35-day intervention (children/adolescents), smartphone monitoring increased PA and reduced weight [61, 67]. Yet, in a large RCT of 365 young adults with overweight/obesity, a smartphone self-monitoring app, which utilised evidence-based BCTs, did not change PA or body weight [68]. Likewise in the ATLAS-RCT in adolescent males, a 20-week multi-modal intervention, including smartphone self-monitoring, did not bring greater weight loss or PA levels [69]. The efficacy of mobile phone apps is summarised by a meta-analysis (*n* = 12 studies) reporting a ~ -1kg body weight reduction but no significant difference in PA level [70].

Mobile apps have been examined against paper diary monitoring with somewhat conflicting results. In a post-hoc analysis of a 6-month RCT of 96 overweight men and women, 6 months mobile app self-monitoring was reported to increase exercise self-monitoring and PA and reduce BMI to a greater extent  than non-app users. [71] Carter et al. reported the greatest mean weight change in 128 overweight volunteers at 6 months with a smartphone app compared with a diary and a website group [72]. Yet, a separate RCT of mobile app PA and diet monitoring highlighted that whilst the intervention caused weight loss, PA level actually reduced [73]. Likewise, a primary care RCT (*n* = 212) interrogating the smartphone self-monitoring app MyFitnessPal reduced weight but did not alter PA level with significant decline in use after 1 month noted [74].

Even though dietary intake and PA are monitored concurrently in all studies, evaluation of their relative impacts on weight loss is challenging; self-monitoring mobile apps likely predominantly affect behaviour change through dietary modification rather than increased PA. Akin to the data in wearables, supporting data for apps is strongest in the short to medium term, with limited evidence interrogating long-term efficacy [70, 75]. Unlike wearables, evidence-based BCTs are not commonly utilised by mobile phone apps which may partly explain their heterogeneous effects on PA [76].

## Short Message Service (Table 3)

**Table 3:** Additional E-health intervention studies and its impact on physical activity level and body weight

Paper	Details	Intervention	Study groups	Findings	Conclusion
*SHED-IT* Morgan et al*.* [113]	*N* = 65, males BMI: ~ 30.6 Age: ~ 35.6	6 months Internet self-monitoring diet/PA with ongoing feedback	**2 groups** Control: face-to-face session + program booklet Intervention: face-to-face session + internet support + self-monitoring	Control: + 1302 steps/day*, − 1881 kJ/day*, − 3.5 kg* Intervention: + 983 steps/day, − 3642 kJ/day* − 5.3 kg* (*p < 0.05 vs baseline)	Both groups lost significant weight vs baseline. Internet trended towards losing more weight than control, with this being driven by DM
Wang et al*. *[82]	*N* = 67, 91%, female BMI: ~ 31.0 Age: ~ 48.2 y	6 weeks Fitbit activity monitor + mobile app ± SMS PA prompts	**2 groups** Control: Fitbit activity monitor + mobile app Intervention: Fitbit activity monitor + mobile app + SMS PA prompts	Control: − 433 steps/day, + 4.3 min/day MVPA Intervention: + 24 steps/day, − 1.1 min/day MVPA	Fitbit activity monitor increased MVPA; the addition of SMS PA prompts did not increase PA long-term vs wearable alone
FITNET Valle et al*. *[94]	*N* = 86, 91% female, cancer survivors BMI: ~ 28.7 Age: ~ 31.7 y	12 weeks Facebook-based intervention boosting MVPA	**2 groups** Control: Facebook-based self-help group Intervention: Facebook-based intervention promoting MVPA	Control: + 55.0 min/week MVPA, + 20.6 min/week LPA, − 0.1 kg Intervention: + 67.9 min/week, MVPA*, + 97.8 min/week LPA*, − 2.0 kg (*p < 0.05 vs control)	Facebook-based PA intervention increased PA and reduced weight in young cancer survivors; however, this was not greater than the control group
*MOBILE POD* Turner-McGriev et al. [97]	*N* = 96, 75%, female BMI = ~ 32.6 Age: ~42.9 y	6 months Podcast ± mobile app for diet + PA monitoring ± Twitter support	**2 groups** Control: BCT podcast Intervention: BCT podcast + mobile app + twitter support	Control: + 96.7 kcal EE, − 242.5 kcal/day EI, − 2.7% weight Intervention: + 86.8 kcal EE, − 288.8 kcal/day EI, − 2.7% weight	Podcast BCT promotes weight loss, but addition of mobile app + twitter support does not have additional benefit
Patel et al*. *[86•]	*N* = 361, uncontrolled T2D, 39.6% female BMI: ~ 37.1 Age; ~ 52.5 y	12 months Wearable device + smart scale ± gamification	**4 groups (all get wearable + smart scale)** Control Game support Game collaboration Game competition	Control: − 214 steps/day, − 1.7 kg Support: + 302 steps/day*, − 3.7 kg Collaboration: + 135 steps/day, − 3.3 kg Competition: + 326 steps/day*, − 2.9 kg (*p < 0.05 vs control)	In patients with uncontrolled T2D, gamification intervention increased PA over 1 year but did not significantly reduce weight vs control

Original table created by authors

*BMI* body mass index, *y* years, *N* number, *PA* physical activity, *MVPA* moderately vigorous physical activity, *p* p-value, *SBWL* standard behavioural weight loss

Short Message Service (SMS) has been utilised as an eHealth intervention whereby individuals receive text messages encouraging behaviour changes including PA [77, 78]. In a 12-month cluster RCT of 250 women, SMS reinforcement utilised alongside a SBWL program resulted in a small difference in weight change between the intervention and control groups at 12 months (− 1.1 kg) with beneficial changes in PA and diet [79]. In an RCT of 52 college students, an 8-week multi-modal intervention including Facebook and Facebook plus SMS demonstrated that the addition of SMS behavioural advice achieved significant weight loss (~ 2.4 kg) [80]. Yet, when implemented in 170 individuals with obesity daily SMS incorporating PA feedback increased step count by ~ 3000 steps/day but did not lead to weight loss. This trial did report significant attrition potentially confounding findings [81]. Similarly, Wang et al. demonstrated the combination of SMS exercise prompts and wearables did not increase PA more than wearables alone [82].

Overall, a meta-analysis including only 6 studies showed that the weighted mean change in body weight with SMS intervention was − 2.56 kg, although importantly both PA and dietary modification were concurrently evaluated and there was a lack of long-term data [83]. Future studies should assess the impact of incorporating BCTs by utilising accelerometry-determined PA data to provide personalised SMS. Whether SMS PA interventions are particularly relevant to older individuals or those from countries where smartphones are less readily available is also unknown. More research is required before SMS PA interventions can be implemented within a healthcare environment.

## Exergaming (Table 3)

Exergaming is a videogame requiring body movements and is available across many systems including traditional consoles and smartphones. It is an innovative intervention which has considerable potential to promote PA across all age groups and in particular younger demographics [8, 84, 85, 86•]. Using accelerometry, it has been shown that when children exergame they are physically active 50% of the time, with 20% of time in moderate-vigorous physical activity and 30% in light PA [85]. Indeed, one 2-year intervention study of 261 school children demonstrated that exergaming proved equivalent to in-person PE classes in stimulating accelerometry-defined PA [87].

The technology has even shown promise in adult populations, with 10 weeks of combined exergaming and activity monitoring increasing PA more than activity monitoring alone [88]. In a 12-week RCT of 40 participants, a mobile phone exergame was shown to bring higher PA levels relative to control with no difference in body weight reported [89]. In addition, college students partaking in regular exercise expend greater energy than when exergaming [90]. In contast, 12 weeks of exergaming in 37 overweight girls did not significantly improve accelerometry defined PA [91].

Overall, the evidence indicates that whilst exergaming has potential to improve PA, there is a paucity of data evaluating exergaming as a weight loss strategy [84]. Future studies need to investigate the long-term efficacy of exergaming for PA promotion and weight reduction. A key question to answer is whether exergaming replaces sedentary screen time or displaces an individual’s usual sports/PA. If the latter, then exergaming would be best promoted in the most sedentary individuals [13].

## Social Networking Sites (Table 3)

Social networking sites (SNS) have been interrogated for their utility in promoting PA and weight loss [92–94]. An example of this is Facebook or the sports SNS Strava, which allows exercisers to document their PA and monitor other users/friends’ progress. SNS provide a unique social interface whereby individuals can be influenced by their network’s positive health behaviours without direct contact [92, 95–97]. The effects are mediated at least partly by the power of social influence [95].

In the FITNET study of 86 cancer survivors, a 12-week SNS PA intervention increased light PA and brought greater weight loss than control (− 2.0kg) [94]. Whilst in the ManUp RCT of 301 middle aged male participants, an IT-based intervention (Web and Mobile App) including SNS support was as effective in improving PA and dietary behaviours as print-based methods [96]. Similarly, Pope et al. reported that an SNS intervention increased PA to similar degrees as a wearable activity monitor. Importantly, participants found the wearable challenging to use potentially biasing results [98, 99].

Overall, whilst SNS interventions may be a useful PA stimulus, data is based on multi-modal interventions making evaluation of their effect on PA and body weight impossible. Most studies have focused on Facebook; future studies should target more recently developed SNS platforms including Instagram, TikTok, WhatsApp and Snapchat. These SNSs generally appeal to differing demographics than Facebook and may prove more successful. Studies must also isolate how to maximise engagement in SNS health interventions and how the intervention could be utilised in the healthcare system [8, 100, 101]. Table 3 provides further studies evaluating SNS, exergaming and SMS interventions.

## Internet-Based Interventions (Table 3)

A variety of internet-based interventions have been evaluated for their ability to promote PA and weight loss including emails, website self-monitoring, smart-scales and podcasts [102–104]. In a 12-week RCT, a weight loss podcast providing diet and PA advice demonstrated significant efficacy in improving MVPA and weight versus control [104]. In the SHED-IT RCT of 65 male University staff and students with overweight, an Internet-based weight-loss program with PA monitoring brought  significantly more weight loss at 6 months vs control (-5.3kg vs -3.5kg). The weight loss was driven by dietary modification, with no change in PA noted [103]. A common theme in many of these studies is that whilst interventions like smart-scales, email counselling and website self-monitoring might promote weight loss, this seems to be achieved predominantly via dietary modification with weak evidence to support internet-based interventions achieving weight loss independently by increasing PA.

## Future Directions

### Design of Future eHealth Intervention Studies

There is considerable scope to design robust eHealth intervention trials to inform the development of a strong evidence base and facilitate integration of eHealth technologies into healthcare. Factors to consider include (i) the independent interrogation of interventions (dietary vs PA), (ii) a focus on body fat distribution rather than simple weight change, (iii) demonstration of long-term efficacy and of weight loss maintenance, (iv) assessment of emerging eHealth interventions and (v) provision of real-world data in a healthcare service.

Factorial trial designs will facilitate quantification of the individual effect of eHealth interventions on PA and body weight. Given that data has demonstrated eHealth interventions reduce central obesity, research should assess the impact of eHealth interventions on ectopic fat depots in the liver, skeletal muscle and heart, given their pathophysiological link with T2D, non-alcoholic fatty liver disease (NAFLD) and cardiovascular disease (CVD) [105]. Long-term data will allow determination of whether PA behaviour changes are sustained beyond 12 months with a dearth of evidence for their efficacy beyond this time point. Maintenance of weight loss is currently a field where eHealth interventions have not been rigorously evaluated; yet, weight regain is common problem so novel approaches are required [16, 106]. Future trials should also interrogate the efficacy of emerging internet-based PA platforms that provide workouts linked to wearable devices and include Apple Fitness, Fiit and programmes provided via YouTube. Health services must also ensure that the populations at greatest need, such as the socio-economically deprived and older individuals (who have greater risk of obesity), have access to eHealth interventions. Finally, trials should focus on creating real-world data of eHealth interventions within health services. Ryu et al. showed that an eHealth intervention linked to electronic patient records was effective in the short term [107•]. Long-term data must be analysed in a similar intervention to demonstrate efficacy. This development will allow closer monitoring of patient progress, highlighting early attrition and consequently facilitating more intensive therapies when necessitated.

### Potential Developments in eHealth Interventions

There is considerable scope for future eHealth innovations considering evolution of artificial intelligence. For example, data-driven feedback from wearables and smartphones may encourage attainment of personalised PA goals via digital personal assistants like Apple Siri or Google Now and digital health coaching (using evidence-based BCTs). Furthermore, there is potential to remotely monitor heart rate using wearable devices to guide physical activity goals, providing real-time feedback to individuals regarding time in MVPA. Additionally, behavioural economics could be incorporated into developing programmes whereby participants are rewarded for increased PA. A lottery could be implemented, whereby individuals are motivated to maintain a behaviour due to the chance of winning. This has potential to reduce attrition and improve adherence to eHealth interventions [23, 108].

## Conclusions (Fig. 1)

**Fig. 1:**
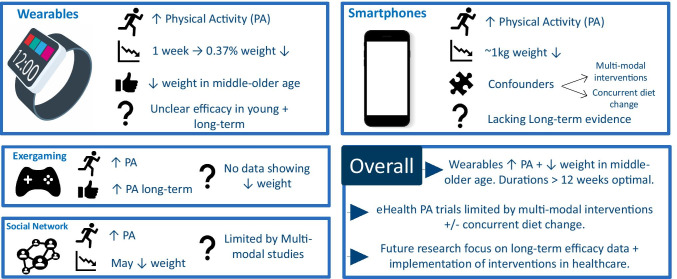
Graphical summary of review findings. Original figure created by authors. PA = physical activity

Overall eHealth interventions provide a novel approach to promote PA and weight reduction. Figure 1 provides a graphical summary of the review’s findings. Wearable technologies may increase PA and promote weight loss in middle to older aged adults with durations of > 12 weeks being optimal, although the duration of effect is uncertain, with little/no evidence beyond 12 months. Smartphone interventions promote weight loss, but due to complex trial designs it is unclear whether this is driven by PA or dietary modification. Exergaming, SNS programmes and SMS reduce weight in some cases, yet there is a lack of long-term data and it is unclear whether PA modification is the mechanistic driver or rather dietary change. Altogether, much more research is needed, particularly with longer-term efficacy data, to demonstrate significant and convincing effects on physical activity patterns or on body weight before eHealth interventions can be implemented within healthcare services. However, it would appear clear that technology will become an asset for health care in the twenty-first century and opportunities exist to make best use of it.

## Abbreviations

SBWL: Standard behavioural weight loss
PA: Physical activity
WL: Weight loss
T2D: Type 2 diabetes mellitus
NALFD: Non-alcoholic fatty liver disease
BMI: Body mass index
BCT: Behaviour change techniques
RCT: Randomised controlled trial
MVPA: Moderate-vigorous physical activity
LPA: Light physical activity
eHealth: Electronic health
